# *In vitro* Fermentation of Digested Milk Fat Globule Membrane From Ruminant Milk Modulates Piglet Ileal and Caecal Microbiota

**DOI:** 10.3389/fnut.2020.00091

**Published:** 2020-07-09

**Authors:** Caroline Thum, Wayne Young, Carlos A. Montoya, Nicole C. Roy, Warren C. McNabb

**Affiliations:** ^1^Food Nutrition & Health Team, AgResearch, Grasslands Research Centre, Palmerston North, New Zealand; ^2^Riddet Institute, Massey University, Palmerston North, New Zealand; ^3^High-Value Nutrition National Science Challenge, Auckland, New Zealand

**Keywords:** milk fat globule membrane, ileal and caecal fermentation, caprine, bovine, ovine

## Abstract

Lipids in milk are secreted as a triacylglycerol core surrounded by a trilayer membrane, the milk fat globule membrane (MFGM). This membrane, known to have important roles in infant brain and intestinal development, is composed of proteins, glycoproteins, and complex lipids. We hypothesized that some of the beneficial properties of MFGM are due to its effects on the gastrointestinal microbiota. This study aimed to determine the effect of a commercial phospholipid concentrate (PC) and enriched bovine, caprine, and ovine MFGM fractions on ileal and hindgut microbiota *in vitro*. Digestion of PC and MFGMs was conducted using an *in vitro* model based on infant gastric and small intestine conditions. The recovered material was then *in vitro* fermented with ileal and caecal inocula prepared from five piglets fed a commercial formula for 20 days before ileal and caecal digesta were collected. After each fermentation, samples were collected to determine organic acid production and microbiota composition using 16S rRNA sequencing. All substrates, except PC (5%), were primarily fermented by the ileal microbiota (8–14%) (*P* < 0.05). PC and caprine MFGM reduced ileal microbiota alpha diversity compared to ileal inoculum. Caprine MFGM increased and PC reduced the ileal ratio of firmicutes:proteobacteria (*P* < 0.05), respectively, compared to the ileal inoculum. Bovine and ovine MFGMs increased ileal production of acetic, butyric, and caproic acids compared to other substrates and reduced the proportions of ileal proteobacteria (*P* < 0.0001). There was a limited fermentation of bovine (3%), caprine (2%), and ovine (2%) MFGMs by the caecal microbiota compared to PC (14%). In general, PC and all MFGMs had a reduced effect on caecal microbiota at a phylum level although MFG source-specific effects were observed at the genus level. These indicate that the main effects of the MFGM in the intestinal microbial population appears to occur in the ileum.

## Introduction

Milk fat is secreted from the mammary gland in the form of milk fat globules (MFG) composed of a triacylglycerol core covered by a trilayer membrane, the milk fat globule membrane (MFGM). The MFGM is a source of bioactive proteins, glycoproteins, and complex lipids known to improve body (1), brain (1–4), immune (5), and intestinal development (6, 7).

MFG are digested and absorbed in different areas of the intestinal tract, which affects the nutritional and functional role of the MFGM components. Initially, it was thought that MFGM components were digested and absorbed in the small intestine (8, 9). Recent studies, however, indicate that specific components of the bovine MFGM, such as glycoproteins, could reach the large intestine (10). *In vitro* digestion of MFGM components has mainly been tested using adult conditions (time, pH, and enzyme concentration) (11, 12). The infant's gastrointestinal tract is immature with suboptimal pH for digestive enzymes and lower concentrations of bile acids and enzymes (13, 14). This could reduce the digestion of MFGM components in the stomach and small intestine and, therefore, increase the amount of undigested MFGM reaching the large intestine.

Digestion of MFGM is affected by the MFGM structure and composition (15). Human and ruminant MFGMs share the same structure, but their different protein and lipid composition may affect digestion and functionality. For instance, proteomic analysis has identified 312, 554, 175, and 140 proteins in human, bovine, caprine, and ovine MFGM, respectively, of which only 87 proteins were common among these species (16, 17). Differences in the profile of complex lipids among MFGMs have also been reported. Phosphatidylcholine, phosphatidylethanolamine, and sphingomyelin, the three major constituents of MFGM polar lipids, account for 62–80% of the total phospholipids in human and bovine MFGM and 90% in ovine and caprine MFGMs (18–22). Thus, differences in MFGM composition may lead to different rates of digestion (15), absorption, and subsequently, different portions of the MFGM reaching the lower small intestine (ileum) prior to being released into the large intestine, where they are available for microbial fermentation.

The effects of a commercial bovine MFGM concentrate, known as phospholipid concentrate (PC), on large intestinal mucosa, microbial profile, and organic acid concentration were recently demonstrated using animal models (6, 23, 24). Bhinder et al. (6), using a “pup-in-a-cup” rat model (5–15 post-natal days of age), show that addition of PC to infant formula restored small and large intestinal growth, microbial composition, Paneth and goblet cell numbers, and tight-junction protein patterns to conditions found in pups fed maternal milk. Another study (24) shows that a combination of PC and prebiotics improved small (duodenum, jejunum, ileum) and large intestinal (ascending colon) maturation (e.g., greater enzymatic activity) and reduced opportunistic bacteria (*Escherichia/Shigella, Klebsiella*) in piglets compared to those fed a control formula.

Recent studies demonstrate that the ileum harbors a significant population of microbes (25) and is associated with variable retention times (72–392 min in healthy individuals) (26), which may have an important role on fermenting undigested food components prior to the large intestine (27). It has been reported, for example, that close to 30% of the digesta entering the ileum of pigs fed a humanized diet was fermented in the ileum (27). In another study, 80% of soluble kiwifruit fiber was fermented in the upper gastrointestinal tract of pigs (28), and this occurred mainly in the ileum. In human adults, a considerable degree of fiber fermentation has also been reported in the upper gastrointestinal tract [47% for pectin (29), 66% for a resistant starch (30)]. Therefore, we hypothesized that dietary bovine, caprine, and ovine MFGMs differently modulate the ileal and caecal microbiota population, resulting in different fermentation end products.

**Figure 1:**
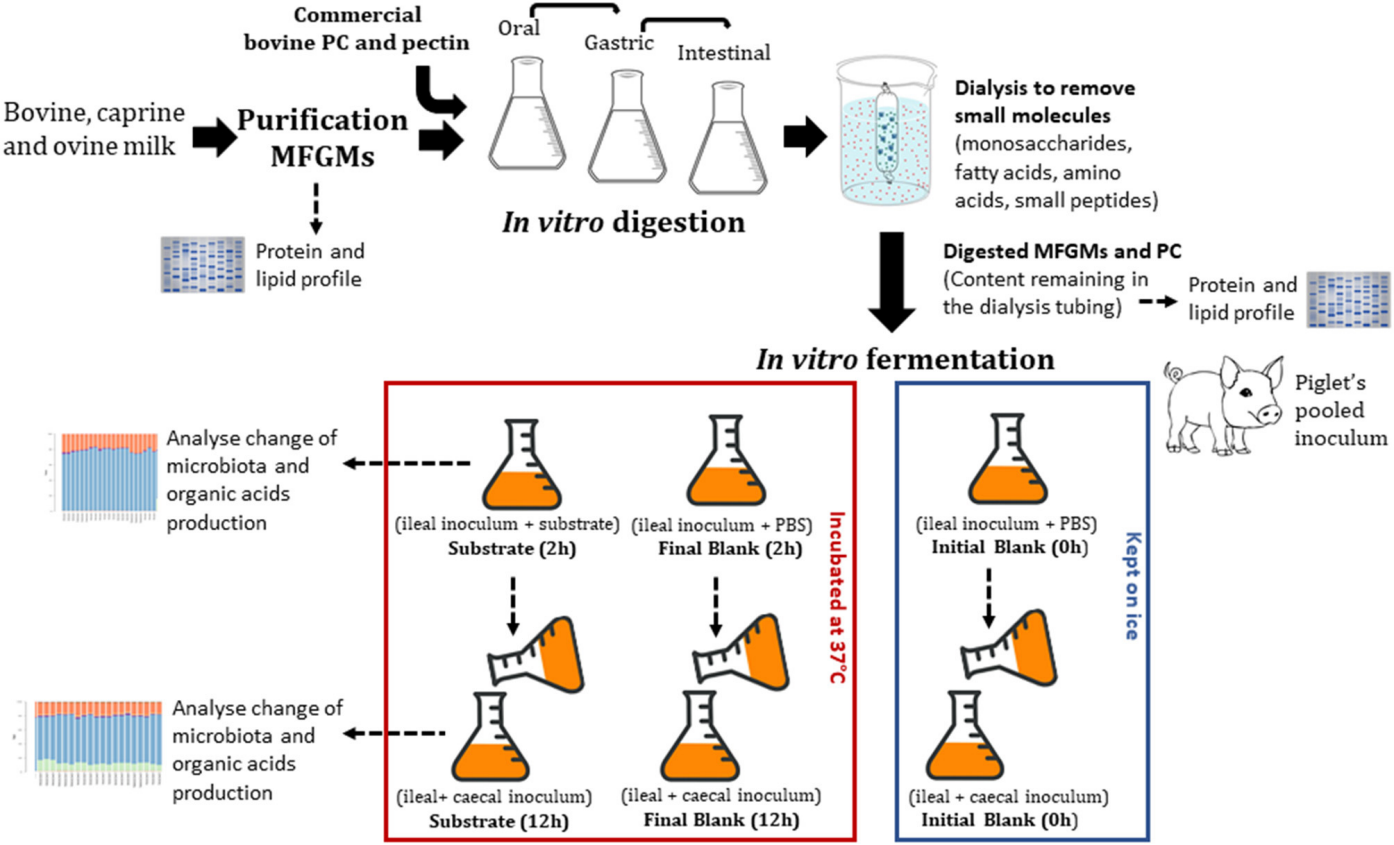
Study design.

This study aimed to determine the effect of PC and enriched bovine, caprine, and ovine MFGM fractions on ileal and caecal microbiota and production of organic acids *in vitro*. *In vitro* gastrointestinal digestion of PC and MFGMs was conducted using conditions that modeled those in infants. The undigested material was then recovered and fermented *in vitro* with ileal inoculum (ileal fermentation) followed by caecal (proximal colonic fermentation) inoculum of formula-fed piglets. After each fermentation, samples were collected to determine organic acid production and microbial profile using 16S rRNA gene amplicon sequencing (Figure 1).

## Materials and Methods

### Milk and Milk Fat Globule Membrane Enrichment Method

A commercial bovine PC product was donated by Tatua Co-operative Dairy Company Ltd., New Zealand. Tatua's PC was obtained by microfiltration of the liquid stream derived from the production of anyhydrous milk fat. Raw, non-homogenized bovine, caprine, and ovine milk were collected from local farms (Palmerston North, New Zealand) and refrigerated at 4°C until processing.

MFGM was enriched according to the method previously described (31) with some modifications. The raw milk was centrifuged at 3,500 × *g* for 15 min at 4°C and the cream layer collected. The cream layer was washed once using phosphate-buffered solution (4.3 mM Na_2_HPO_4_, 2.7 mM KCl, 1.8 mM KH_2_PO_4_, and 137 mM NaCl) followed by tri-distilled water (twice for bovine milk and only once for caprine and ovine milk). Each washing step was followed by centrifugation at 4,500 × *g* for 20 min at 4°C. Tri-distilled water was added to the cream sample (1:1 v/v) and stirred for 10 min at 50°C in a water bath. The mixture was then sheared using a kitchen mixer (Kenwood Kitchen machine, KMC 510, 1000W, Auckland, NZ), for 10 min at speed 2 to release the fat from the cream. The fat fraction was separated from the MFGM material by centrifugation at 12,000 × *g* for 30 min at 4°C. After removing the fat fraction and aqueous phase, the pellet was freeze-dried and stored at −20°C.

### Digestion

MFGM and PC were digested following the *in vitro* static Infogest model (32) with modifications (pH, enzyme concentration, and time) to represent those found in 5-months-old infants (14). Simulated digestion fluids [such as simulated salivary fluids (SSF), simulated gastric phase solution, and small intestinal phase solution (SIF)] were made up of the corresponding electrolyte stock solutions [containing KCl, KH_2_PO_4_, NaHCO_3_, NaCl, MgCl_2_(H_2_O)_6_, and (NH_4_)_2_CO_3_], enzymes, CaCl_2_, and water as described in Minekus et al. (32).

#### Oral Phase

Dry MFGM or commercial PC treatment samples (5 g) were mixed with 3.5 mL of SSF (pH 7) (32). Salivary α-amylase solution (0.5 mL, 960 U/mL α-amylase from human saliva Type XIII-A; A1031, Sigma, St. Louis, MO, USA, made up in SSF) was added, followed by 25 μL of 0.3 M CaCl_2_ and water to a final volume of 10 mL. All reagents were pre-warmed to 37°C before being mixed with MFGM or PC samples. Immediately after mixing, the gastric phase digestion was conducted as milk is swallowed without mastication.

#### Gastric Phase

The total oral digested sample was mixed with 7.5 mL of SGF (pH 6) (32), 1 mL porcine pepsin (2,000 U/mL; P6887, Sigma, made up in SGF), 1 mL gastric lipase (800 U/mL; 62305, Sigma, made up in SGF), 5 μL of 0.3 M CaCl_2_, and water to a final volume of 20 mL. The digestion solution was incubated at 37°C for 1.5 h with the first hour at pH 6 followed by pH 5.

#### Intestinal Phase

The total gastric digested sample was neutralized by adjusting the pH to 6.5. The neutralized sample was then mixed with 11 mL of SIF (32), 2.5 mL of bile salts stock (16 mM; B8756, Sigma), 40 μL of 0.3 M CaCl_2_, and water to a final volume of 40 mL. The solution was incubated for 10 min at 37°C with shaking. Pancreatin solution (5.0 mL, 800 U/mL, based on trypsin activity (P1750, Sigma, made up in SIF) was then added to the solution and incubated at 37°C for 2 h. Digestion was stopped by putting the samples on ice prior to dialysis. The small intestinal sample was then dialyzed against deionized water (Spectra, New Brunswick, USA, CE Membrane 100–500 MWCO 31 mm width, 20 mm diameter) for 24 h at 4°C. After dialysis, the samples remaining inside the dialysis tubes (i.e., undigested fraction) were freeze-dried and stored at −20°C. Dialysis is often used as a simulation model for the absorption of free amino acids and small peptides (di- or tripeptides) in the small intestine (10). After *in vitro* digestion and dialysis, the recovered material was calculated as:

$$\begin{array}{l} Recovery\;material\;\left(\%\right)=\frac{\left({\left(C_{i}-C_{f}\;\right)}^{*}100\right)}{C_{i}}, \end{array}$$

where *C*_*i*_ and *C*_*f*_ are the amounts of digested material added to the dialysis tube and recovered after dialysis, respectively.

### Fermentation

All procedures involving animals were approved by the Grasslands Animal Ethics Committee under the New Zealand Animal Welfare Act 1999 (AEC#12997). Five male piglets 11 days old were used. During acclimatization (4 days), piglets were housed together and fed a bovine-based infant formula. Thereafter, piglets were housed individually for 16 days. The bovine-based infant formula (51.3% protein, 36.2% fat, 5.2% ash, 1.5% moisture) (Fonterra, Palmerston North, New Zealand) was provided *ad libitum* every 4 h during the whole study. On post-natal day 31 (11 days old on arrival and 20 days of study), piglets were euthanized and ileal (last 30 cm of the small intestine) and caecal digesta were collected in plastic bags previously flushed with CO_2_ (to maintain anaerobic conditions) and kept on ice until fermentation (27).

The pig ileal and caecal inocula were prepared in an anaerobic cabinet. Ileal and caecal digesta were pooled (to reach the volume necessary to ferment all substrates) in similar amounts between piglets and homogenized (1:5, w/v) with a sterilized and anaerobic phosphate buffer saline solution (PBS, 0.1 M + 0.5 g/L cysteine) at pH 7 for ileum and pH 6.5 for caecum. The initial pH (pH 7 for ileum and pH 6.5 for caecum) was determined by measuring the pH in the animal ileum and caecum at the time digesta samples were collected. The pH at the end of the fermentation was not determined. Inoculum was filtered through sterilized layers of cheesecloth and stirred constantly. Ileal inoculum (5 mL) was added first to sterilized McCartney bottles containing 5 mL PBS (blank) or 5 mL PBS containing 100 mg of freeze-dried substrate (digested bovine, caprine, or ovine MFGMs, PC, or undigested citrus pectin). Citrus pectin (Spectrum Chemical, New Brunswick, USA), a highly fermentable dietary fiber, was used as a fermentation control (Figure 1). Pectin was not *in vitro* digested previously to the fermentation as mammalian enzymes do not cleave pectin. Thus, citrus pectin (100 mg) was fermented directly with the ileal inoculum.

Blanks (inoculum and PBS) were divided into initial and final blank. PBS was used in blanks as a high concentration of ileal and caceal digesta has been shown to provide nitrogen and minerals required by the microbiota (27). Initial blanks were kept on ice until processing, and final blanks were incubated at 37°C for 2 h for ileal or 12 h for caecal fermentation (Figure 1).

Twelve bottles per blank (initial and final) or per substrate were used (three technical replicates for each analysis). After 2 h of ileal fermentation, half of the bottles (*n* = 6) were removed from the incubation and kept on ice to reduce microbial activity. The remaining bottles were inoculated with the caecal inoculum using a pipette (5 mL, 1:5, w/v in PBS); initial caecal fermentation blanks were kept on ice or otherwise fermented for an additional 12 h (Figure 1). Half of the bottles (blanks and substrates) were placed in an autoclave (121°C for 20 min) to completely inactivate microbial fermentation and to remove the end products of organic matter fermentation (e.g., SCFA) before determination of fermentability. The other half of the bottles were thoroughly mixed, and an aliquot (1 mL) was collected into Eppendorf tubes. The Eppendorf tubes were centrifuged (14,000 × *g* for 15 min at 4°C) and the supernatant (0.5 mL) collected and stored at −20°C for subsequent determination of organic acids. The precipitate was used to determine the composition of the microbial population.

Organic matter fermentability of the MFGM substrates was calculated accordingly to Montoya et al. (33). In short,

$$\begin{array}{l} \text{Organic matter }{\text{fermentability}}_{in\;vitro}\;(\%)\\ \;=\frac{\frac{\left({\text{OM}-(\text{OM}}_{\text{a}}-{(\text{OM}}_{\text{blank initial}}+{\text{OM}}_{\text{blank final}}\text{)}\right)}{2}}{{\text{OM}}_{\text{b}}}\times 100, \end{array}$$

where OM_b_ and OM_a_ are the amounts of organic dry matter in the substrate (undigested MFGM) either before or after *in vitro* fermentation. OM_blank initial_, OM_blank final_ are the amounts of OM in the blank bottles (which contained inoculum but no substrate) before (initial) and after (final) *in vitro* fermentation, respectively.

### Chemical Composition Analysis

#### Proteins

Samples of MFGMs and PC before and after digestion were resuspended in water and protein concentration determined by Qubit Protein Assay (ThermoFisher, Auckland, New Zealand). The protein concentration in bovine, caprine, and ovine MFGMs and PC before and after *in vitro* digestion was adjusted to 1.6 mg/mL. The samples were further diluted (1:1) in 2 × Laemmli sample buffer (5% β-mercaptoethanol) (Bio-Rad Laboratories, Inc., Hercules, CA, USA), denatured by heating at 95°C for 5 min, and centrifuged at 2,500 × *g* for 30 min at 4°C. Samples (10 μL) with a total protein concentration of 8 μg were then loaded onto SDS-PAGE gels (4–15% Mini-PROTEAN^®^ TGX™ Precast Protein Gels, Bio-Rad Laboratories). A molecular weight (MW) marker (Precision Plus Protein Unstained Standards 3.5–260 kDa; Bio-Rad Laboratories) was used. The gels were electrophoresed in a mini-protein system (Bio-Rad Laboratories) at 110 V using a Bio-Rad power supply unit for 50 min. The SDS gels were stained for 1 h by Coomassie Brilliant Blue R-250 staining solution (Bio-Rad Laboratories) and destained overnight. Putative identification of proteins was conducted based on the estimated MW of the bands and the MW reported for each protein in previous studies for bovine (34, 35), caprine (36), and ovine (16) MFGMs. To visualize glycoproteins, other SDS-PAGE gels were loaded as described above but using a glycoprotein MW marker (CandyCane™) (ThermoFisher P21857) and a total protein concentration of 5 μg. Glycoproteins were stained using Pro-Q^®^ Emerald 300 Glycoprotein Gel and Blot Stain Kit (ThermoFisher P21857) as per supplier instructions.

#### Lipid Profile

Lipid extraction and phospholipid analysis were conducted as previously described (37, 38). Total fatty acid analysis was conducted by rinsing the samples of MFGM and PC before and after digestion with 1:1 chloroform methanol and drying prior to methylation. The samples were then methylated in a two-stage process. First, 1.5 mL of 0.5 M NaOH in methanol was added to the samples and incubated for 10 min at 80°C. Second, 2.5 mL of boron trifluoride complex in methanol (10%) was added to the samples and incubated for 30 min at 80°C. After cooling, the fatty acid fraction was extracted by adding 1 mL of iso-octane to the mixture and vortexed, followed by 5 mL of saturated NaCl, which was mixed by shaking for 30 s by hand. The sample was centrifuged (500 × *g* for 10 min) and the iso-octane layer transferred to a vial. The extraction was repeated a second time with 1 mL of iso-octane and combined with the first fraction. Samples (1 μL) were injected onto a Polar FAME (Restek RTX 2330 column, 105 m × 0.25 mm i.d, 0.20 μm film thickness; Restek Corporation, Bellefonte, USA), and fatty acid profiles were analyzed by gas chromatography (GC).

### Organic Acid Detection

The preparation of samples for organic acid analysis was a two-step procedure. In the first step, organic acids were extracted into an aqueous solution for GC Flame Ionization Detection (GC-FID). GC-FID analysis of acetic, butyric, propionic, valeric, *iso*-valeric, *iso*-butyric, and caproic acids were conducted (39). The second step was an ether extraction followed by derivatization with N-methyl-N-t-butyl-dimethyl-silyl-trifluoroacetamide (Sigma-Aldrich) for GC analysis of lactic, formic, and succinic acids. These latter three organic acids have to be derivatized because formic acid responds poorly to FID, and lactic and succinic acids both have low volatility and high polarity (40). In short, 100 μL concentrated HCl and 800 μL diethyl ether were mixed vigorously with 200 μL of acidified fermented medium fluid supernatant containing the internal standard, 2-ethyl butyric acid (W242918, Sigma-Aldrich). After allowing the mixed sample to settle for 1 min, the top ether layer was transferred into a 2-mL vial. This extraction process was repeated by adding a further 800 μL diethyl ether to the aqueous phase of the first vial. The two extracts were then combined. The extract (800 μL) was derivatized with 100 μL of derivatization reagent with N-methyl-N-t-butyl-dimethyl-silyl-trifluoroacetamide and heated in a crimp-top GC vial for 20 min at 80°C. Samples were left at room temperature for ~48 h to allow for complete derivatization of lactic acid.

The analysis was carried out using a Shimadzu GC-2010 Plus gas chromatogram (Shimadzu Corporation, Kyoto, Japan) with helium ionization detector and a Zebron ZB-5MS 30 m × 0.25 mm I.D. × 0.25 μm film capillary column. A split injection of a 1 μL sample was made at a ratio of 20:1 with a column helium flow rate of 21 mL/min. Injector and detector temperatures were both 240°C. The column temperature was initially held at 50°C for 2 min and then increased at 5°C/min to 130°C, followed by 15°C/min to 240°C (held for 4.7 min).

### Microbial Profile

Microbial DNA was extracted from pellets using Machey Nagel NucleoSpin Soil kits (Macherey-Nagel, Duren, Germany) following the manufacturer's instructions with the addition of a bead-beating step using a BioSpec Mini-Beadbeater 96 set to 4 min and 0.1 mm silica beads (BioSpec, Bartlesvile, USA). DNA samples were analyzed by 16S rRNA gene amplicon sequencing using the Illumina MiSeq Platform with 2 × 250 bp paired-end sequencing. Primers targeting the V3 and V4 region of the 16S rRNA gene were used for amplification as follows: forward primer: 5′-TCGTCGGCAGCGTCAGATGTGTATAAGAGACAGCCTACGGGNGGCWGCAG; reverse primer: 5′-GTCTCGTGGGCTCGGAGATGTGTATAAGAGACAGGACTACHVGGGTATCTAATCC.

PCR thermal cycler conditions were used as specified in the Illumina library preparation protocol (95°C for 3 min; 25 cycles of 95°C for 30 s, 55°C for 30 s, 72°C for 30 s; 72°C for 5 min; hold at 4°C; Illumina 2015). Sequence reads were paired and quality trimmed using Qiime 1.9 (41) with the default parameters except for the following: chimeric sequence removal and OTU picking was performed using Usearch61 and taxonomy assigned against the Silva 128 small subunit ribosomal RNA database. Following quality trimming, the median number of reads was 29,295 with a minimum of 13,019, maximum of 51,579, and a standard deviation of 9,315. Alpha diversity was assessed using Faith's phylogenetic diversity indices and differences between means analyzed by ANOVA.

### Statistical Analysis

Overall microbial communities were compared using permutation multivariate analysis of variation (MANOVA) using distance matrices as implemented using the adonis function in the vegan package for R (42). Comparisons of relative abundances for individual taxa were performed by permutation ANOVA using the aovp function in the lmPerm package (43) for R. *P*-values were adjusted for multiple testing using the Benjamini–Hochberg false discovery rate (FDR) method. Fisher's LSD test was used for *post-hoc* pairwise analysis.

A two-way ANOVA model was used to determine the effects of MFGMs/PC (*n* = 4), inoculum (*n* = 2), and the interaction on OM fermentability using the Proc Mixed of the statistical software SAS. For organic acid production, a one-way ANOVA model was used. The model diagnostics for each response variable were tested using the ODS graphics procedure and the repeated statement of SAS. The repeated statement was used to test for homogeneity of variances by fitting models with the restricted maximum likelihood method and comparing using the log-likelihood ratio test. The selected model for each response variable had adjusted equal variances across treatments. When the *F* value of the model was significant (*P* ≤ 0.05), the means were compared using the adjusted Tukey test.

## Results

### Milk Fat Globule Purification and Composition

**Figure 2:**
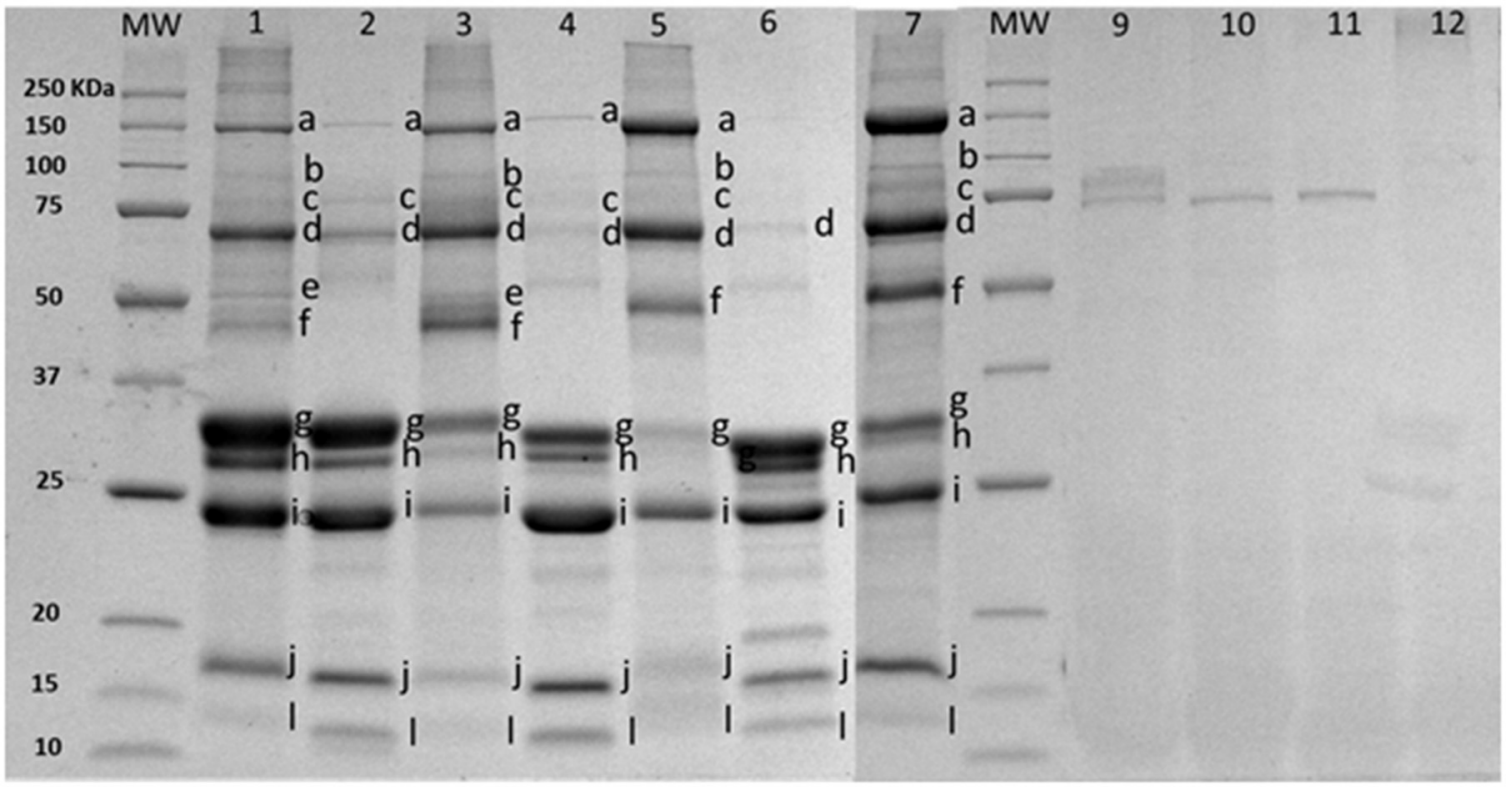
SDS-PAGE pattern of milk fat globule membrane (MFGM) proteins in milk and MFGM purified before and after digestion. MW, molecular weight marker (KDa). The MFGM proteins are from phospholipid concentrated (lane 1), bovine milk (lane 2), bovine MFGM (lane 3), caprine milk (lane 4), caprine MFGM (lane 5), ovine milk (lane 6), ovine MFGM (lane 7), material remaining after digestion for bovine MFGM (lane 9), caprine MFGM (lane 10), ovine MFGM (lane 11), and phospholipid concentrated (line 12). Proteins were identified accordingly with MW as (a) xanthine oxidase (~145 kDa), (b) PAS III (~100 kDa), (c) CD36 (~75 kDa), (d) butyrophilin (64 kDa bovine, 67 kDa caprine), (e) or (f) adipophilin (50–52 kDa) or lactadherin (PAS 6/7; 52–58 kDa bovine, 55 kDa caprine), (g) α-caseins (~22–25Kda), (h) β-caseins (~23–24 kDa), (i) κ-caseins (~19 kDa), (j) β-lactaglobulin (~18 kDa), and (l) α-lactalbumin (~14 kDa).

The enrichment method increased the concentration of proteins putatively identified in Figure 2 as part of the MFGM [mucin 1 (250–450 kDa), xanthine oxidase (band a), PAS III (band b), CD36 (band c), butyrophilin (band d), adipophilin and/or lactadherin (PAS 6/7; 52–58 kDa bovine, 55 kDa caprine) (band e or f), compared to bovine (lanes 2 and 3), caprine (lanes 4 and 5), and ovine lanes 6 and 7)] milk. Skim milk proteins (caseins, bands g–i) and whey proteins (β-lactoglobulin, α-lactalbumin, band j and l) were observed in all MFGM samples after enrichment. Based on band intensity, a higher proportion of caseins was observed in commercial PC (lane 1) compared to purified bovine MFGM (lane 3). Total protein concentration in PC and purified bovine, caprine, and ovine MFGMs were 45.1, 25.3, 22.7, and 22.2%, respectively (Table 1).

**Table 1:** Protein and lipid composition (%) of phospholipid concentrated (PC) and purified milk fat globule membrane from bovine, caprine, and ovine before and after infant simulated *in vitro* digestion.

	**Before digestion**				**After digestion**			
	**PC**	**Bovine**	**Caprine**	**Ovine**	**PC**	**Bovine**	**Caprine**	**Ovine**
Total protein	45.1	25.3	22.7	22.2	3.2	4.2	4.5	6.3
Total lipids	14.1	36.1	14.5	30.3	12.5	33.0	10.9	11.8
Total polar lipids	5.22	4.24	5.83	3.06	2.13	1.78	1.05	1.16
Phosphatidylinositol	0.29	0.25	0.47	0.27	0.12	0.16	0.09	0.11
Phosphatidylethanolamine	1.15	0.92	0.66	0.06	0.03	0.12	0.01	0
Phosphatidylserine	1.06	0.51	1.52	0	0	0	0	0
Phosphatidylcholine	1.69	1.52	1.92	1.29	0.24	0.48	0.13	0.2
Sphingomyelin	1.03	1.04	1.25	1.44	1.74	1.01	0.82	0.85

After *in vitro* digestion and dialysis, the recovered material (content remaining in the dialysis tube) was 59, 35, 15, and 47% of the original PC, bovine, goat, and sheep MFGM, respectively. The SDS-PAGE pattern of MFGM after digestion showed only a small number of uncharacterized protein bands with a MW similar to PAS III and CD36 in bovine, ovine, and caprine MFGM samples but not in PC (Figure 2, lanes 10–13, respectively). The total protein in the recovered digested MFGM was 3.2, 4.2, 4.5, and 6.3% for PC, bovine, caprine, and ovine, respectively (Table 1).

**Figure 3:**
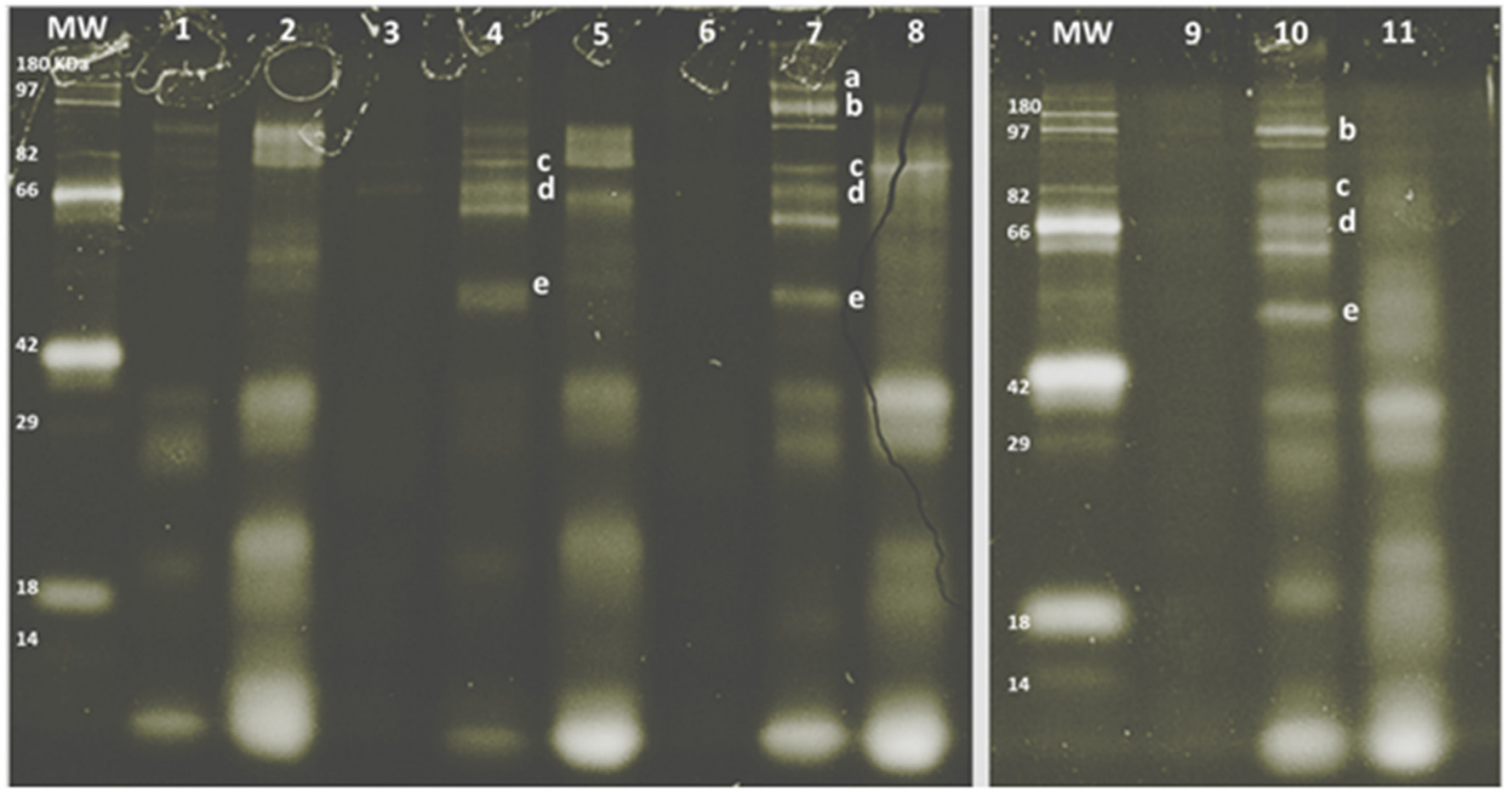
SDS-PAGE pattern of milk fat globule membrane (MFGM) glycoproteins in milk and MFGM purified before and after digestion. MW, molecular weight marker. The MFGM proteins are from phospholipid concentrate (lane 1), digested phospholipid concentrate (lane 2), bovine milk (lane 3), bovine MFGM (lane 4), digested bovine MFGM (lane 5), caprine milk (lane 6), caprine MFGM (lane 7), digested caprine MFGM (lane 8), ovine milk (lane 9), ovine MFGM (lane 10), and digested ovine MFGM (line 11). Proteins were identified accordingly with MW as (a) xanthine oxidase (~145 kDa), (b) PAS III (~100 kDa), (c) CD36 (~75 kDa), (d) butyrophilin (64 kDa bovine, 67 kDa caprine), (e) lactadherin (PAS 6/7; 52–58 kDa bovine, 55 kDa caprine).

The SDS-PAGE pattern of glycoproteins in milk, enriched MFGM samples, and PC (before and after digestion) are shown in Figure 3. Bands were putatively identified, based on MW, as xanthine oxidase, PAS III, CD36, butyrophilin, and lactadherin. After gastrointestinal digestion, a large proportion of glycoproteins were observed (lanes, 2, 5, 8, and 11), especially with lower MW than the parent glycoproteins observed in the intact MFGM (lanes, 1, 4, 7, and 10).

The total polar lipid content in PC, bovine, caprine, and ovine MFGM was 5.2, 4.2, 5.8, and 3.0%, respectively (Table 1). Phosphatidylcholine was the most concentrated phospholipid in PC (1.7%), bovine (1.5%), and caprine (1.9%) enriched MFGM and sphingomyelin was for ovine MFGM (1.4%). For all samples, the concentration of polar lipids was reduced after digestion to 1.0–2.1% of total lipids.

Palmitic acid (C16) and the fatty acids C18:1c9, C:18, and C14 were the most abundant fatty acids in PC (23, 29, 12, and 8%), bovine (29, 16, 17, and 11%), caprine (32, 19, 16, and 9%) and ovine (25, 20, 17, and 8%) MFGM fractions, respectively (Table 2). Digestion of PC, bovine, caprine, and ovine MFGM fractions had a small effect (≤ 10% variation) on the proportions of the fatty acids C18:2n6, C15:0, C20:3n6, C9 t11-CLA, iso C17, iso C16, C20:0, anteiso C17, C17:1, and C16:0. In contrast, a reduction of ≥65% on the proportion of the medium chain fatty acids (MCFA) (C10:0, C12:0) and C14:1 was observed after the digestion of all samples.

**Table 2:** Fatty acid profile (% of total fatty acids^a^) of phospholipid concentrated (PC) and purified milk fat globule membrane (MFGM) from bovine, caprine, and ovine before and after infant simulated *in vitro* digestion.

**Substrate**	**Before digestion**				**After digestion**			
	**PC**	**Bovine**	**Caprine**	**Ovine**	**PC**	**Bovine**	**Caprine**	**Ovine**
C10:0	1.84	2.45	5.60	4.05	0.18	0.28	0.40	0.36
C12:0	3.44	3.21	3.25	2.65	1.06	0.73	0.66	0.51
C14:0	8.65	11.16	9.34	8.31	6.28	7.71	5.47	4.54
iso C14	0.00	0.10	0.00	0.08	0.00	0.05	0.00	0.00
C14:1	0.45	0.43	0.07	0.06	0.16	0.10	0.00	0.00
C15:0	0.82	1.16	0.94	0.94	0.77	1.07	0.84	0.81
iso C15	0.16	0.33	0.14	0.21	0.15	0.25	0.11	0.15
Anteiso C15	0.34	0.46	0.20	0.34	0.28	0.33	0.13	0.22
C16:0	23.28	29.28	32.17	25.91	25.12	30.11	34.74	25.74
iso C16	0.17	0.25	0.25	0.21	0.17	0.26	0.27	0.22
C16:1	1.33	0.67	0.34	0.41	1.10	0.45	0.24	0.32
C17:0	0.44	0.67	0.83	0.83	0.51	0.73	1.02	0.93
iso C17	0.58	0.67	0.49	0.74	0.59	0.70	0.55	0.80
Anteiso C17	0.43	0.45	0.36	0.44	0.45	0.47	0.42	0.51
C17:1	0.30	0.22	0.21	0.20	0.32	0.17	0.19	0.20
C18:0	12.69	17.25	16.98	17.75	14.08	19.33	22.31	21.25
C18:1 c11	1.03	0.68	0.64	0.73	1.17	0.86	0.83	0.98
C18:1 t11	2.91	5.90	0.82	4.49	3.36	7.34	1.11	6.30
C18:1 t9	0.26	0.19	0.29	0.26	0.35	0.31	0.44	0.40
C18:1c9	28.93	16.08	19.47	20.89	32.61	20.49	23.62	27.15
C18:2 n6	2.74	0.68	2.15	1.04	2.54	0.78	1.85	1.06
C18:3 n3	0.99	0.40	0.43	0.58	0.76	0.34	0.25	0.37
C20:0	0.17	0.23	0.35	0.29	0.17	0.26	0.51	0.37
C20:1 8	0.00	0.03	0.00	0.06	0.00	0.00	0.00	0.00
C20:3n6	0.29	0.06	0.00	0.04	0.28	0.10	0.00	0.00
C20:4 n6	0.39	0.10	0.20	0.13	0.32	0.12	0.17	0.17
C20:5 n3	0.00	0.04	0.00	0.04	0.00	0.00	0.00	0.00
C22	0.22	0.19	0.20	0.15	0.25	0.26	0.28	0.23
C22:1	0.00	0.03	0.00	0.00	0.00	0.00	0.00	0.00
C22:2	0.33	0.08	0.08	0.10	0.20	0.10	0.20	0.17
C22:5	0.58	0.13	0.40	0.31	0.52	0.19	0.27	0.31
C22:6 n3	0.08	0.00	0.12	0.09	0.00	0.00	0.00	0.00
C24:0	0.13	0.14	0.11	0.12	0.17	0.16	0.15	0.18
C24:1	0.00	0.00	0.00	0.00	0.00	0.00	0.00	0.00
C9 t11-CLA	1.21	0.76	0.26	1.44	1.22	0.81	0.00	1.01
MCFA	5.28	5.66	8.85	6.69	1.24	1.01	1.06	0.87
LCFA	87.35	87.52	86.67	84.98	91.52	92.32	95.07	92.48
VLCFA	2.55	1.33	1.16	2.21	2.35	1.51	0.90	1.91
SFA	53.35	67.99	71.20	63.02	50.23	62.71	67.85	56.82
UFA	41.82	26.52	25.48	30.86	44.88	32.14	29.18	38.44
MUFAs	35.21	24.27	21.84	27.10	39.05	29.71	26.43	35.35
PUFAs	5.40	1.49	3.39	2.32	4.62	1.62	2.74	2.08

*MCFA, medium-chain fatty acids (C6-10). LCFA, long-chain fatty acids (C13-21). VLCFA, very long-chain fatty acids (C22 or higher). SFA, saturated fatty acids. UFA, unsaturated fatty acids. MUFAs, mono-unsaturated fatty acids. PUFAs, polyunsaturated fatty acids*.

a *The results for the fatty acid profile analysis are in % fatty acid but do not add up to 100% as unidentified fatty acid were observed in the samples*.

### Ileal and Caecal Fermentation

There was a significant interaction between the location of fermentation (i.e., inoculum) and the substrate (i.e., digested MFGMs) on the organic matter fermentability (*P* < 0.05; Table 3). All substrates, except PC, were primarily fermented by the ileal microbiota (*P* < 0.05). As expected, pectin had the highest ileal and caecal fermentability (28 and 31%, respectively, *P* < 0.05) compared to bovine (ileal, 7.7% and caecum, 11.2%), caprine (8.8 and 10.6%), ovine (14.0 and 16.2%), MFGMs and PC (5.5 and 19.3%).

**Table 3:** *In vitro* ileal and caecal organic matter (OM) fermentability of digested bovine, caprine, and ovine milk fat globule membrane (MFGM); phospholipid concentrate (PC); and pectin substrates in piglets.

**Substrate**	**PC^1^**		**Bovine^1^**		**Caprine^1^**		**Ovine^1^**		**Pectin^1^**			***P*****-value**		
**Location**	**Ileum**	**Caecum**	**Ileum**	**Caecum**	**Ileum**	**Caecum**	**Ileum**	**Caecum**	**Ileum**	**Caecum**	**SEM**	**Location (L)**	**Substrate (S)**	**L × S**
OM fermentability %	5.5^e^	19.3^bc^	7.7^de^	11.2^cd^	8.8^de^	10.6^cde^	14.0^cd^	16.2^cd^	27.7^ab^	30.5^a^	1.8	<0.001	<0.001	0.030

1 *Ileal fermentation (2 h) was conducted with the undigested substrates prior to caecal fermentation. Means without a common letter differ, P < 0.05. SEM, Standard Error of the Mean*.

**Figure 4:**
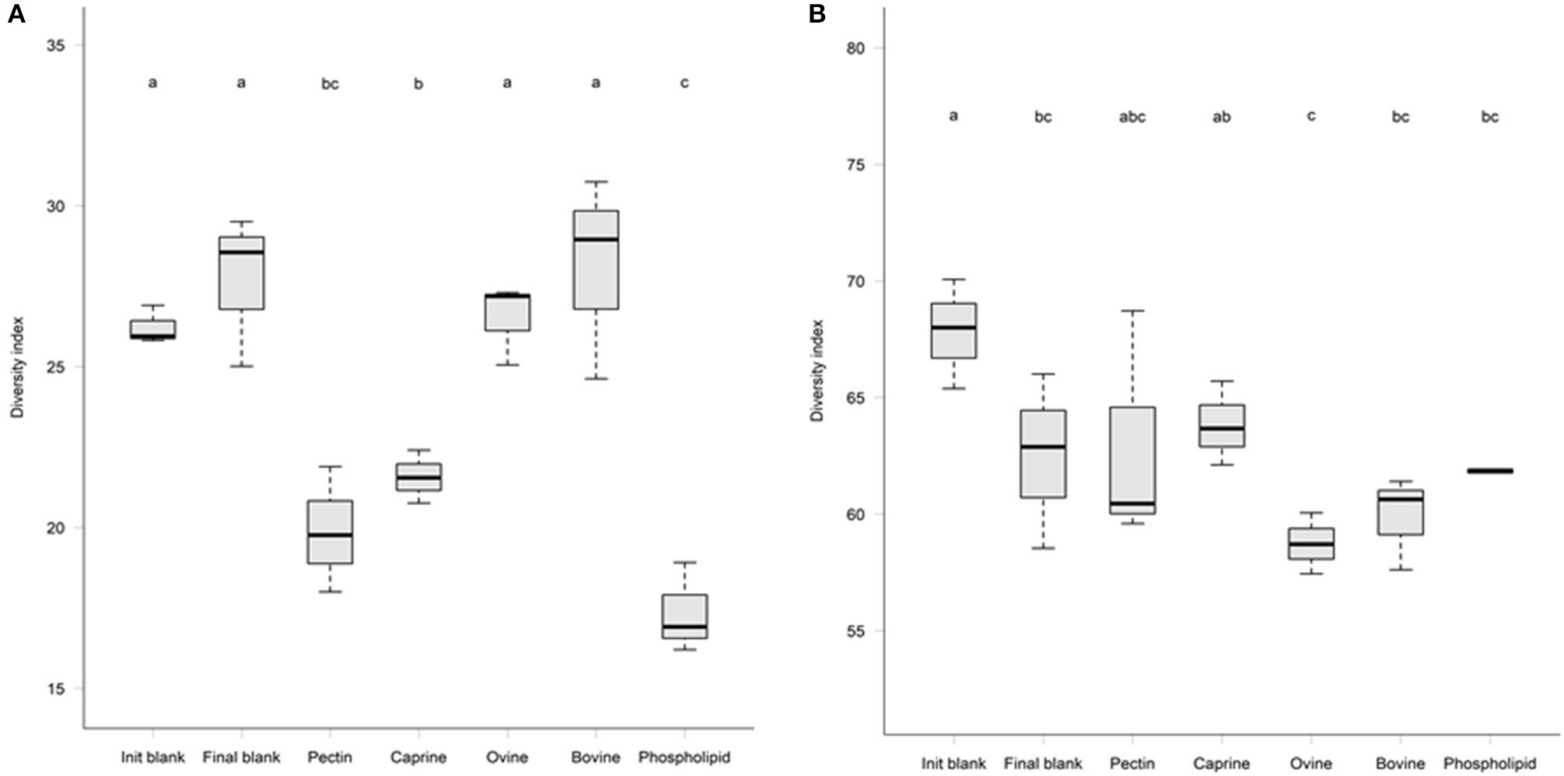
Microbial alpha-diversity after 2 h *in vitro* ileal fermentation **(A)** followed by 12 h of *in vitro* caecal fermentation **(B)** of undigested bovine, caprine, ovine milk fat globule membrane (MFGM), and phospholipid concentrate (PC) in piglets. Pectin was used as a fermentation control. Values are presented as mean ± standard deviation. Means within each without a common letter differ, *P* ≤ 0.05.

After 2 h of ileal microbiota incubation at 37°C, a decrease in the proportions of Fusobacteria was observed in the final blank compared to the initial blank (Table 4). At the genus level, higher unclassified *Peptostreptococcaceae* and lower proportions of *Turicibacter* and *Fusobacterium* genera were observed in the final blank compared to the initial blank (Table 5). Incubation of digested ovine and bovine MFGM fractions for 2 h had no effect on ileal microbial alpha diversity (Figure 4A) but increased the firmicutes:proteobacteria ratio (Table 4) compared to the initial and final blanks.

**Table 4:** Relative abundances of bacteria at the phylum level for the piglet's ileal inoculum (initial blank) and after 2 h of incubation alone (final blank) or with bovine, caprine, and ovine MFGM; phospholipid concentrate (PC); and pectin substrates.

**Phyla**	**Initial blank^1^**	**Final blank^1^**	**PC^1^**	**Bovine^1^**	**Caprine^1^**	**Ovine^1^**	**Pectin^1^**	***P*-value**
Firmicutes	74.2 ± 1.3^c^	77.5 ± 0.6^b^	74.6 ± 0.7^c^	79.9 ± 2.3^ab^	80.8 ± 0.2^a^	79.6 ± 0.9^ab^	78.3 ± 2.7^ab^	0.002
Proteobacteria	22.5 ± 1.1^a^	20.2 ± 0.8^b^	24.3 ± 0.8^a^	17.1 ± 1.4^c^	17.5 ± 0.2^c^	18.0 ± 0.7^bc^	20.1 ± 2.4^b^	<0.001
Fusobacteria	2.3 ± 0.2^a^	1.2 ± 0.2^bc^	0.6 ± 0.01^c^	1.8 ± 0.9^ab^	0.9 ± 0.1^c^	1.2 ± 0.1^bc^	1.0 ± 0.3^c^	0.006
Actinobacteria	0.31 ± 0.07^bc^	0.37 ± 0.04^ab^	0.15 ± 0.05^d^	0.47 ± 0.12^a^	0.41 ± 0.02^ab^	0.48 ± 0.03^a^	0.25 ± 0.09^cd^	<0.001
Bacteroidetes	0.25 ± 0.03^ab^	0.24 ± 0.01^ab^	0.04 ± 0.01^c^	0.27 ± 0.07^a^	0.07 ± 0.006^c^	0.20 ± 0.002^b^	0.078 ± 0.03^c^	<0.001
TM7	0.11 ± 0.03^bc^	0.17 ± 0.03^ab^	0.04 ± 0.007^d^	0.17 ± 0.04^ab^	0.12 ± 0.002^bc^	0.20 ± 0.05^a^	0.10 ± 0.04^cd^	0.003
Firmicute:Proteobacteria	3.30 ± 0.24^de^	3.83 ± 0.20^cd^	3.07 ± 0.13^e^	4.70 ± 0.51^a^	4.62 ± 0.09^a^	4.41 ± 0.24^ab^	3.94 ± 0.65^bc^	<0.001

1 *Values are presented as mean ± standard deviation. Means within each phylum without a common letter differ, P ≤ 0.05*.

**Table 5:** Relative abundances of significant bacteria classified to the lowest identified taxonomic level in the piglet's ileal inoculum (initial blank) and after 2 h of incubation alone (final blank) or with bovine, caprine, and ovine MFGM; phospholipid concentrate (PC); and pectin substrates.

**Taxa^1^**	**Initial blank^2^**	**Final blank^2^**	**PC^2^**	**Bovine^2^**	**Caprine^2^**	**Ovine^2^**	**Pectin^2^**	***P*-value**
Unclassified *Peptostreptococcaceae* (f)	22.6 ± 0.6^d^	27.1 ± 0.7^a^	21.6 ± 0.5^d^	25.5 ± 0.1^bc^	26 ± 0.3^ab^	25.7 ± 0.8^bc^	24.6 ± 0.7^c^	<0.001
*Turicibacter* (g)	15.7 ± 0.8^b^	11.9 ± 0.9^e^	17.1 ± 0.7^a^	12.3 ± 0.5^e^	11.8 ± 0.2^e^	12.6 ± 1.5^de^	14.2 ± 0.4^c^	<0.001
*Escherichia*/*Shigella* (g)	11.4 ± 0.7^b^	10.7 ± 0.8^bc^	12.9 ± 0.4^a^	9.0 ± 0.3^e^	9.1 ± 0^e^	9.6 ± 0.4^de^	10.3 ± 0.1^cd^	<0.001
Unclassified *Enterobacteriaceae* (f)	6.8 ± 0.3^ab^	5.9 ± 0.6^bcd^	7.4 ± 0.1^a^	4.6 ± 0.9^e^	4.9 ± 0.1^de^	5.1 ± 0.3^de^	5.7 ± 1.2^cd^	<0.001
*Streptococcus* (g)	5.2 ± 0.3^bc^	5.9 ± 0.2^bc^	4.8 ± 0.4^c^	6.8 ± 0.8^ab^	7.8 ± 0.4^a^	5.9 ± 1.5^bc^	5.1 ± 1.8^bc^	0.010
*Fusobacterium* (g)	2.1 ± 0.2^a^	1.0 ± 0.1^bc^	0.6 ± 0.0^c^	1.5 ± 0.8^ab^	0.8 ± 0.1^c^	1.0 ± 0.0^bc^	0.9 ± 0.2^c^	0.005
*Enterobacter* (g)	2.0 ± 0.1^a^	1.6 ± 0.1^ab^	2.2 ± 0^a^	1.4 ± 0.2^b^	1.1 ± 0^b^	1.3 ± 0.1^b^	2.2 ± 0.9^a^	0.002
Unclassified Clostridiales (o)	1.8 ± 0.2^abc^	2.0 ± 0.1^a^	1.2 ± 0.1^d^	2.0 ± 0.3^ab^	1.6 ± 0^bc^	1.8 ± 0.2^abc^	1.6 ± 0.2^c^	0.005
*Veillonella* (g)	0.4 ± 0^ab^	0.5 ± 0^a^	0.2 ± 0^c^	0.3 ± 0.1^bc^	0.4 ± 0^bc^	0.5 ± 0^ab^	0.3 ± 0.1^bc^	0.004
*Lactobacillus* (g)	0.4 ± 0.1^bc^	0.5 ± 0.1^ab^	0.3 ± 0^c^	0.5 ± 0.1^ab^	0.6 ± 0^a^	0.5 ± 0^ab^	0.4 ± 0.1^abc^	0.050
*Prevotella* (g)	0.09 ± 0.02^a^	0.09 ± 0.01^a^	0.01 ± 0.01^b^	0.09 ± 0.04^a^	0.02 ± 0.01^b^	0.07 ± 0.01^a^	0.02 ± 0.01^b^	0.001
*Rothia* (g)	0.24 ± 0.05^bc^	0.27 ± 0.01^abc^	0.12 ± 0.05^d^	0.36 ± 0.08^a^	0.32 ± 0.03^ab^	0.36 ± 0.02^a^	0.20 ± 0.09^cd^	<0.001

1 *Taxa identified to the lowest rank; (g) genus, (f) family, (c) class, (o) order*.

2 *Values are presented as mean ± standard deviation. Means within each bacterial taxon without a common letter differ, P ≤ 0.05*.

PC and caprine MFGMs reduced the ileal microbial alpha diversity compared to blanks and bovine and ovine MFGMs (Figure 4A). Although PC maintained the ratio of firmicutes:proteobacteria to the levels observed in the initial blank (Table 4), caprine MFGM increased the ratio of firmicutes:proteobacteria compared to the initial and final blanks (Table 4). In general, PC reduced the proportions of unclassified *Peptostreptococcaceae*, unclassified Clostridiales, *Veillonella, Lactobacillus, Prevotella*, and *Rothia* and increased the proportions of unclassified *Enterobacteriaceae, Turicibacter*, and *Escherichia*/*Shigella* compared to the other substrates and the final blank (Table 5). In general, caprine MFGM increased *Streptococcus* and decreased *Prevotella* genera compared to both blanks and other substrates. Bovine, caprine, and ovine MFGMs decreased the proportions of *Escherichia*/*Shigella* genera and unclassified *Enterobacteriaceae* compared to the final blank.

A higher concentration of acetic acid was produced by ileal microbiota fermenting ovine MFGM, whereas higher concentrations of butyric and caproic acids were produced when fermenting bovine and ovine MFGMs (Table 6) compared to other substrates. Formic and lactic acid production did not differ between the different types of digested MFGMs (*P* > 0.05).

**Table 6:** Organic acid production (mmol/kg incubated substrate) after *in vitro* ileal fermentation of digested bovine, caprine, and ovine milk fat globule membrane (MFGM); phospholipid concentrate (PC); and pectin (control substrate).

**Organic acid^3^**	**Substrate^1^**					**SEM**	***P*-value^2^**
	**PC**	**Bovine**	**Caprine**	**Ovine**	**Pectin**		
Acetic	0.3^b^	0.0^b^	0.0^b^	1.0^a^	0.0^b^	0.1	<0.001
Butyric	0.0^b^	33.0^a^	0.0^b^	35.6^a^	0.0^b^	0.9	<0.001
Caproic	0.0^c^	12.0^a^	0.0^c^	15.2^a^	0.0^c^	0.3	<0.001
Formic	30.7^a^	25.2^a^	29.4^a^	30.7^a^	5.3^b^	2.0	<0.001
Lactic	126.4^a^	121.5^a^	121.6^a^	126.8^a^	23.3^b^	2.5	<0.001

1 *Values are means and pooled standard error of the mean (SEMs) for each organic acid, n = 3 for each substrate and organic acid combination*.

2 *Means within each organic acid without a common letter differ, P ≤ 0.05. Organic acids succinic and propionic acid were not detected in the ileal fermentation*.

3 *Concentration substrate from values found in the final blank*.

Incubation of the caecal inoculum (initial blank) for 12 h reduced the alpha-diversity (final blank) (Figure 4B) and changed microbial composition as observed in the principal coordinates analysis (PCoA; Figures S1–S3). An increased firmicutes:proteobacteria ratio was observed in the final blank compared to the initial blank (Table 7). At the genus level, higher unclassified *Peptostreptococcaceae*, unclassified *Lachnospiraceae, Clostridium, Oscillibacter, Turicibacter, Caprococcus*, and unclassified Clostridia genera and lower proportions of *Escherichia*/*Shigella*, unclassified Ruminococcaceae, *Prevotella, Megasphaera, Bacteroides*, unclassified Prevotellaceae, and *Fusobacterium* genera were observed in the final blank compared to the initial blank (Table 8).

**Table 7:** Relative abundances of bacteria at the phylum level in the piglet's caecal microbiota inoculum (initial blank) and after 12 h of incubation alone (final blank) or with bovine, caprine, and ovine MFGM; phospholipid concentrate (PC); and pectin substrates.

**Phyla**	**Initial blank^1^**	**Final blank^1^**	**PC^1^**	**Bovine^1^**	**Caprine^1^**	**Ovine^1^**	**Pectin^1^**	***P*-value**
Firmicutes	61.6 ± 1.0^b^	70.1 ± 1.6^a^	67.0 ± 0.8^a^	66.8 ± 4.8^a^	67.7 ± 0.6^a^	67.0 ± 0.8^a^	69.2 ± 3.6^a^	0.04
Proteobacteria	17.3 ± 0.3^ab^	15.5 ± 0.4^bc^	16.9 ± 0.2^abc^	17.2 ± 1.8^ab^	15.1 ± 1.1^c^	18 ± 0.5^a^	16.5 ± 1.4^abc^	0.06
Bacteroidetes	16.3 ± 0.8^a^	10.2 ± 0.9^b^	11.2 ± 1.1^b^	11.1 ± 2^b^	11.7 ± 0.1^b^	10.1 ± 0.7^b^	10.3 ± 1.6^b^	0.005
Fusobacteria	2.0 ± 0.4^abc^	1.2 ± 0.1^c^	1.9 ± 0^abc^	2.1 ± 0.7^ab^	2.4 ± 0.5^a^	2.2 ± 0.3^a^	1.3 ± 0.4^bc^	0.06
Other	0.92 ± 0.04^abc^	0.8 ± 0.07^c^	1.07 ± 0.01^ab^	1 ± 0.15^ab^	1.1 ± 0.11^a^	0.96 ± 0.13^abc^	0.88 ± 0.06^bc^	0.04
Verrucomicrobia	0.39 ± 0.1^a^	0.12 ± 0.02^b^	0.13 ± 0.01^b^	0.08 ± 0.02^b^	0.13 ± 0.01^b^	0.06 ± 0.03^b^	0.08 ± 0.03^b^	0
Actinobacteria	0.35 ± 0.02^bc^	0.59 ± 0.06^a^	0.30 ± 0.01^c^	0.42 ± 0.04^b^	0.36 ± 0.02^bc^	0.34 ± 0.03^bc^	0.41 ± 0.10^bc^	0.003
Spirochaetes	0.35 ± 0.05^c^	0.38 ± 0.012^bc^	0.47 ± 0.008^abc^	0.49 ± 0.09^b^	0.46 ± 0.07^abc^	0.54 ± 0.01^a^	0.37 ± 0.10^c^	0.031378
Synergistetes	0.31 ± 0.01^c^	0.45 ± 0.009^ab^	0.57 ± 0.02^a^	0.41 ± 0.15^bc^	0.43 ± 0.03^bc^	0.47 ± 0.06^ab^	0.47 ± 0.04^ab^	0.0396
TM7	0.13 ± 0.02^b^	0.15 ± 0.02^b^	0.18 ± 0.04^ab^	0.14 ± 0.02^b^	0.22 ± 0.01^a^	0.16 ± 0.01^b^	0.13 ± 0.02^b^	0.0284
Euryarchaeota	0.08 ± 0.02^b^	0.14 ± 0.03^a^	0.11 ± 0.01^ab^	0.07 ± 0.03^b^	0.15 ± 0.03^a^	0.07 ± 0.02^b^	0.08 ± 0.02^b^	0.0278
Firmicute:Proteobacteria	3.54 ± 0.003^c^	4.50 ± 0.22^a^	3.96 ± 0.01^abc^	3.92 ± 0.67^abc^	4.49 ± 0.41^a^	3.72 ± 0.10^bc^	4.22 ± 0.56^ab^	0.05

1 *Values are presented as mean ± standard deviation. Means within each phylum without a common letter differ, P ≤ 0.05*.

**Table 8:** Relative abundances of significant bacteria classified to the lowest identified taxonomic level in the piglet's caecal microbiota inoculum (initial blank) and after 12 h of incubation alone (final blank) or with bovine, caprine, and ovine MFGM; phospholipid concentrate (PC); and pectin substrates.

**Taxa^1^**	**Initial blank^2^**	**Final blank^2^**	**PC^2^**	**Bovine^2^**	**Caprine^2^**	**Ovine^2^**	**Pectin^2^**	***P*-value**
Unclassified *Lachnospiraceae* (f)	13.4 ± 1.4^c^	21.2 ± 1.6^ab^	20.7 ± 1.5^ab^	24.2 ± 6.4^a^	22.8 ± 2.0^a^	26.0 ± 2.4^a^	16.9 ± 1.9^bc^	0.001
*Escherichia*/*Shigella* (g)	11.2 ± 0.6^ab^	9.9 ± 0.4^cd^	10.9 ± 0.3^abc^	10.9 ± 0.8^abc^	9.6 ± 1.0^d^	11.8 ± 0.3^a^	10.5 ± 0.7^bcd^	0.02
Unclassified *Ruminococcaceae* (f)	9.5 ± 0.3^a^	7.4 ± 0.6^b^	7.4 ± 0.6^b^	7.0 ± 0.2^bc^	7.6 ± 0.1^b^	6.3 ± 0.3^c^	7.4 ± 0.4^b^	0.0006
Unclassified Clostridiales (o)	8.2 ± 0.2^c^	8.2 ± 0.1^c^	9.2 ± 0.0^a^	8.3 ± 0.2^bc^	8.7 ± 0.1^ab^	8.1 ± 0.5^c^	8.1 ± 0.2^c^	0.005
*Prevotella* (g)	7.3 ± 0.4^a^	4.2 ± 0.2^bc^	3.7 ± 0.6^cde^	3.5 ± 0.4^de^	3.9 ± 0.2^cde^	3.2 ± 0.2^e^	4.7 ± 0.1^b^	<0.0001
*Megasphaera* (g)	4.4 ± 0.4^a^	1.1 ± 0.2^b^	1.4 ± 0.1^b^	1.0 ± 0.1^b^	1.2 ± 0.1^b^	1.0 ± 0.0^b^	1.2 ± 0.1^b^	<0.0001
*Blautia* (g)	4.2 ± 0.1^ab^	4.0 ± 0.3^ab^	3.9 ± 0.2^ab^	3.5 ± 0.6^bc^	3.7 ± 0.5^b^	2.8 ± 0.3^c^	3.6 ± 0.7^bc^	0.02
Unclassified *Enterobacteriaceae* (f)	3.2 ± 0.0^bc^	3.2 ± 0.1^bc^	3.3 ± 0.1^bc^	3.5 ± 0.4^ab^	2.9 ± 0.1^c^	3.5 ± 0.2^ab^	3.4 ± 0.4^bc^	0.04
*Bacteroides* (g)	3.2 ± 0.4^a^	1.6 ± 0.0^c^	2.5 ± 0.1^b^	2.9 ± 0.6^ab^	2.7 ± 0.1^ab^	2.7 ± 0.2^ab^	1.7 ± 0.4^c^	0.0006
Unclassified *Peptostreptococcaceae* (f)	3.1 ± 0.3^d^	5.7 ± 0.9^b^	3.1 ± 0.3^d^	3.7 ± 0.2^cd^	3.6 ± 0.2^cd^	3.0 ± 0.2^d^	8.2 ± 0.7^a^	<0.0001
*Clostridium* (g)	2.1 ± 0.2^cd^	2.9 ± 0.3^b^	1.4 ± 0.1^e^	1.7 ± 0.0^de^	1.8 ± 0.1^de^	1.4 ± 0^e^	4.5 ± 0.5^a^	<0.0001
Unclassified *Prevotellaceae* (f)	2.1 ± 0.4^a^	1.4 ± 0.2^bc^	1.4 ± 0.0^abc^	1.3 ± 0.4^bc^	1.4 ± 0.1^ab^	1.0 ± 0.1^bc^	1.5 ± 0.6^ab^	0.02
*Oscillibacter* (g)	2.0 ± 0.2^d^	3.0 ± 0.2^c^	3.3 ± 0.1^bc^	3.2 ± 0.2^bc^	3.9 ± 0.3^a^	3.6 ± 0.2^ab^	3.3 ± 0.3^bc^	<0.0001
Unclassified Bacteroidetes (p)	1.9 ± 0.0^a^	1.5 ± 0.3^ab^	1.8 ± 0.3^a^	1.8 ± 0.4^a^	1.9 ± 0.1^a^	1.7 ± 0.1^a^	1.2 ± 0.1^b^	0.009
Fusobacterium (g)	1.8 ± 0.4^ab^	1.1 ± 0.1^cd^	1.8 ± 0.0^abc^	1.9 ± 0.7^a^	2.2 ± 0.4^a^	2.0 ± 0.3^a^	1.2 ± 0.4^bcd^	0.004
*Turicibacter* (g)	1.1 ± 0.0^c^	1.7 ± 0.2^a^	0.8 ± 0.0^c^	1.4 ± 0.2^b^	0.9 ± 0.0^c^	1.1 ± 0.1^c^	1.8 ± 0.1^a^	<0.0001
*Lactobacillus* (g)	1.0 ± 0.0^ab^	1.1 ± 0.0^a^	0.7 ± 0.0^c^	0.8 ± 0.1^bc^	0.8 ± 0.1^c^	0.8 ± 0.1^c^	1.0 ± 0.1^ab^	0.003
*Coprococcus* (g)	0.9 ± 0.1^b^	1.3 ± 0.0^a^	1.0 ± 0.0^b^	1.0 ± 0.1^b^	1.0 ± 0.1^b^	0.9 ± 0.1^b^	1.0 ± 0.1^b^	0.001
Unclassified Clostridia (c)	0.6 ± 0.0^c^	1.0 ± 0.0^b^	1.1 ± 0.0^ab^	1.0 ± 0.0^b^	1.0 ± 0.1^b^	1.2 ± 0.1^a^	0.8 ± 0.0^c^	<0.0001
*Barnesiella* (g)	0.6 ± 0.0^ab^	0.5 ± 0.0^abc^	0.6 ± 0.0^a^	0.5 ± 0.1^abc^	0.5 ± 0.1^abc^	0.4 ± 0.0^c^	0.4 ± 0.0^c^	0.05

1 *Taxa identified to the lowest rank; (g) genus, (f) family, (c) class, (o) order, (p) phylum*.

2 *Values are presented as mean ± standard deviation. Means within each bacterial taxon without a common letter differ, P ≤ 0.05*.

In general, after 12 h of incubation with caecal inoculum, PC and all MFGMs had no effect of caecal microbiota diversity (Figure 4B) and composition (phylum; Table 7), and genus (Table 8) compared to the final blank. Some MFG source-specific effects were observed. For instance, ovine MFGM increased the proportions of *Escherichia*/*Shigella, Oscillobacter*, Fusobacteria, Bacteroides genera, and unclassified Clostridia and decreased the proportions of unclassified *Ruminococcaceae, Prevotella, Blautia Lactobacillus*, and *Clostridium* genera compared to the final blank. PC and caprine MFGM increased the proportions of unclassified Clostridiales, whereas caprine MFGM also increased the proportions of *Oscillobacter* and Fusobacteria compared to the final blank. Higher concentrations of butyric acid were found in the caecal fermentation when incubated with ovine and bovine MFGM compared to other substrates (Table 9).

**Table 9:** Organic acid production (mmol/kg incubated substrate) after *in vitro* ileal and caecal fermentation of undigested bovine, caprine, ovine milk fat globule membrane (MFGM); phospholipid concentrate (PC); and pectin (control substrate).

**Organic acid^3^**	**Substrate^1^**					**SEM**	***P*-value^2^**
	**PC**	**Bovine**	**Caprine**	**Ovine**	**Pectin**		
Acetic	178.1^ab^	182.2^a^	172.4^ab^	166.4^b^	151.7^ab^	4.5	0.007
Butyric	25.2^b^	41.5^a^	23.9^b^	40.1^a^	20.0^c^	0.6	<0.0001
Caproic	0.0^c^	9.6^b^	0.0^c^	10.8^a^	0.0^c^	0.1	<0.0001
Valeric	9.8^a^	9.6^ab^	9.4^b^	0.0^c^	9.4^b^	0.05	<0.0001
Succinic	78.7^a^	81.7^a^	81.9^a^	78.3^a^	44.1^b^	1.4	<0.0001

1 *Values are means and pooled standard error of the means (SEMs) for each organic acid, n = 3 for each substrate and organic acid combination*.

2 *Means within each organic acid without a common letter differ, P ≤ 0.05. No differences on propionic, formic, and lactic concentrations were observed after incubation with ileal and caecal digesta*.

3 *Concentration substrates from values found in the final blank*.

## Discussion

In this study, bovine, caprine, and ovine MFGM-enriched fractions were digested with gastric and small intestine enzymes simulating the infant GIT prior to being fermented by ileal and caecal microbiota from piglets fed an infant formula from 11 to 31 days post-natally. As stated in the study's hypothesis, fermentation of MFGM substrates produced organic acids and changed the ileal and caecal microbiota composition in an MFG source-specific manner.

The enriched MFGM fraction obtained in this study contained proteins and polar lipids characteristic of the native MFGM as previously reported for bovine (44), caprine (36), and ovine milk (16). The molecular diversity of lactadherin, for example, was observed in the SDS-PAGE gel with one band identified for caprine and ovine MFGMs and two for bovine MFGM (ranging from 52 to 58 kDa) (45). Higher glycosylation of caprine xanthine oxidase compared to bovine xanthine oxidase may have led to the observation of a xanthine oxidase band in SDS-PAGE gel stained with ProQ Emerald (Figure 3, Lane 7 and band a), which has been described elsewhere (36). The composition of commercial PC was different from bovine MFGM with an increased concentration of total proteins and polar lipids, in particular, caseins and phosphatidylserine. Differences in composition between commercial PC and bovine MFG are likely due to variations in enrichment methodology and composition of starting material (46).

This study is the first to report the digestion of a bovine, caprine, and ovine MFGM-enriched fractions using an *in vitro* model with enzymatic concentrations, time of incubation, and pH adapted to simulate conditions found in 5-months old infants (14). Caprine MFGM had a lower recovery after digestion and dialysis (15%) compared to other substrates (35–49%), probably due to a low concentration of both total protein and total lipids in the purified caprine MFGM.

The SDS-PAGE profiles of PC, bovine, caprine, and ovine MFGMs after digestion were similar to the reported data for bovine MFGM digestion using an adult *in vitro* model (10, 15). Different levels of proteolysis after MFGM digestion may be explained by the MFG source-specific profile and concentration of MFGM proteins, especially glycoproteins, in the start material (16, 17, 45). Indeed, a different band pattern in the ProQ-Emerald stained SDS-PAGE gel was observed after the digestion of MFGMs from different sources (bovine, caprine, and ovine). Previous studies using adult *in vitro* digestive conditions showed that MFGM glycoproteins (i.e., butyrophilin, xanthine oxidase, PAS6/7, and mucins) were hydrolyzed to different extents (9, 10). Mucin 1 had the highest resistance to digestion compared to other glycoproteins (i.e., butyrophilin), and a part of this protein was still detected with the original MW after gastric and small intestine digestion (10). Human milk N-glycans were also shown to resist gastrointestinal digestion and were detected in infant stools (47).

Recently, the INFOGEST 2.0 *in vitro* gastrointestinal food digestion method recommended the use of rabbit gastric lipase due to its similar stereospecificity for TAG hydrolysis when compared to human gastric lipase (48). At the time this study was performed, however, rabbit gastric lipase was not commercially available, and the fungal lipases from *Rhizopus orizae* were used. *R. orizae* lipase was shown to have greater lipolysis rate (46 vs. 10%) and be insensitive to FA chain length compared to human gastric enzyme, which released only C8:0 and C10:0 under both gastric and intestinal conditions (49).

To understand the effect of PC and enriched bovine, caprine, and ovine MFGM fractions on ileal and caecal microbiota and production of organic acids, piglet ileal and cacecal inoculum were used to ferment the material remaining after *in vitro* digestion of PC, bovine, caprine, and ovine MFGM preparations. In these *in vitro* assays, PBS was used to homogenize the microbiota instead of nutritive medium used by some authors (50). The PBS is used when a high concentration of feces is used. This high concentration provides the nitrogen and minerals required by the microbiota (51), which must be provided in a nutritive medium when small amount of feces (or cultures) are used. In all our *in vitro* fecal fermentation studies, we have followed the PBS approach to avoid any confounding effect of adding nutrients in a different amount (or ratio) to that in ileal and hindgut (27).

*In vitro* ileal organic matter fermentation for MFGM substrates showed different fermentability, ranging from 6 to 14%, whereas pectin was higher (28%). Despite the high ileal OM fermentability of pectin, this fermentation produced the lowest concentration of organic acids compared to MFGM and PC. This may indicate fermentation products not measured in this study (e.g., pyruvic acid) could have been produced during pectin fermentation in greater amounts before being converted to other organic acids (e.g., lactic acid) later in the fermentation (52).

In general, Ileal microbiota had substantial proportions of *Turibacter* and *Streptococcus*, which are known lactate producers (53, 54) and may explain the link between ileal lactate and microbiome composition. Bovine and ovine MFGMs produced higher concentrations of acetic, butyric, and caproic acids with limited effects on the ileal microbiota composition. Increase concentration of actinobacteria and bacterioidetes, however, were observed after bovine and ovine MFGM ileal fermentation and may explain the increased concentration of acetic acid observed (55). Butyric acid are mainly produced by members of *Clostridiaceae* family (56), which were also increased after bovine and ovine MFGM fermentation compared to other substrates.

Bovine and ovine substrates reduced the proportions of ileal proteobacteria, which, at elevated levels, have been linked in other studies to dysbiosis (57, 58). Proteobacteria are facultative anaerobes known to consume oxygen, altering the pH and lowering the redox potential, making the intestine suitable for colonization by strict anaerobes. Proteobacteria, in the neonatal small and large intestine, are affected by the type of feeding with a higher frequency of these bacteria in formula-fed infants compared to breast-fed infants [reviewed in (59)].

PC and caprine MFGMs had the largest effect on the ileal microbiota as observed by the reduced alpha diversity, clear separation in the principal coordinate analysis (Figure S1), and changes in the taxonomic composition compared to blanks and other substrates. Differences in composition found after digestion in PC (i.e., lower total protein and increased polar lipid concentration, in particular, sphingomyelin and MCFA) and caprine MFGM (lower total fat and polar lipids, in particular very long-chain fatty acids or VLCFA) may have led to the different effects observed in the ileal microbiota. We speculate that components of caprine MFGM and PC may have benefited more dominant members of the ileal microbiota, which consequently may have caused a decrease of rarer microbial taxa, decreasing alpha diversity. Caprine MFGM, for example, increased the ratio of firmicutes:proteobacteria, whereas PC reduced the proportions of unclassified Clostridiales, *Prevotella*, and/or *Lactobacillus* and increased the proportions of *Enterobacteriaceae* in the ileal microbiota compared to the initial and final blanks. These changes are consistent with the microbial profile observed in the ileal microbiota of piglets fed infant formula compared to piglets fed sow milk (60, 61). The relevance of these findings to human infants, however, still needs to be determined as studies on the ileal microbial in infants are still lacking. In one study, Barret at al. (62) reported that human infant's ileal microbiota is dominated by actinobacteria (94%) up to 40 days of life and then mostly by proteobacteria and firmicutes from 50 to 217 days of life (62).

Fermentation of the organic matter in bovine, caprine, and ovine MFGMs was relatively minor after ileal fermentation (~3% increased after ileal fermentation) although PC was greater (~14% after ileal fermentation). Thus, the total ileal–caecal *in vitro* fermentation ranged from 11% (bovine and caprine MFGM substrates) to 19% (PC). Although lower fermentability of organic matter was observed from the caecal microbiota, production of organic acids was observed for all substrates. This may be explained by (1) products of ileal fermentation (i.e., organic acids, glycans) being used by the caecal microbiota as a carbon source, (2) the presence of a larger array of caecal microbial enzymes that are able to degrade resistant non-dietary components (63, 64), and/or (3) longer time of fermentation. Increased concentration of butyric and caproic acids observed after caecal fermentation of bovine and ovine MFGM substrates may originate from ileal fermentation, which was inoculated with caecal microbiota. In contrast, acetate, succinate, and valeric acids, observed for all substrates, are known to be produced by specific microbes [i.e., *Bacteroides* spp., *Prevotella* spp. (65) and *Clostridium* spp (66)], which were present only in the caecal inoculum in the current study.

This study was not designed to demonstrate which specific component (protein, lipid, glycan) of the enriched MFGM from ruminant milks were responsible after *in vitro* gastrointestinal digestion for the effects observed in the ileal and caecal microbiota. However, changes in the proportions of bacterial genus observed in this study are likely due to the combination of fermentable substrates (e.g., glycans) and antimicrobial components (e.g., linolenic acid, C18:3, and polyunsaturated fatty acids or PUFAs) within the enriched MFGM fraction. After digestion, PC, for example, was enriched in sphingomyelin and unsaturated fatty acids (UFAs) compared to the other substrates. Unsaturated fatty acids with 18 carbon long chains—oleic acid (C18:1), linoleic (C18:2), and linolenic acid (C18:3)—have potent antibacterial activities against Gram-positive bacteria (67, 68). Degradation products of sphingomyelin, such as sphingosine, have shown bactericidal activities against *E. coli* and other pathogenic bacteria (69, 70).

In conclusion, the enriched MFGM fractions from bovine, caprine, and ovine milk contain proteins and polar lipids characteristic of the native MFGM. The material of the enriched MFGM fractions remaining after *in vitro* gastric and intestinal digestion, using infant conditions, were mainly fermented *in vitro* by the ileal microbiota, demonstrating the potential importance of ileal fermentation. Fermentation of the MFGM substrates produced organic acids and changed the proportions of the ileal and caecal microbiota in a MFG source-specific manner. Although this study was not designed to identify specific components of the digested MFGMs responsible for the effects on the ileal and caecal microbiota, they are likely to be a combination of fermentable substrates (glycans) and antimicrobial components of the MFGM. More studies are needed to further understand the effects of infant digestion on MFGM components and the *in vivo* effects of MFGM on the ileal and large intestinal microbiota.

## Data Availability Statement

The datasets generated for this study can be found in NCBI SRA, NCBI Accession No. PRJNA610043.

## Ethics Statement

The animal study was reviewed and approved by Grasslands Animal Ethics Committee under the New Zealand Animal Welfare Act 1999 (AEC#12997).

## Author Contributions

CT, CM, NR, and WM designed the study. WY performed the *in vivo* study. CT and CM performed the *in vitro* study. CT supported by CM and WY analyzed the data and wrote the paper, which was reviewed by NR and WM. All authors contributed to the article and approved the submitted version.

## Conflict of Interest

The authors declare that the research was conducted in the absence of any commercial or financial relationships that could be construed as a potential conflict of interest.
