# Supplementary material for: In vitro Fermentation of Digested Milk Fat Globule Membrane From Ruminant Milk Modulates Piglet Ileal and Caecal Microbiota
Source: Front Nutr. 2020 Jul 9;7:91. doi: 10.3389/fnut.2020.00091 (PMC7363764; doi:10.3389/fnut.2020.00091)
Supplement: Supplementary file 1 [file Data_Sheet_1.docx]

**Supplemental material**


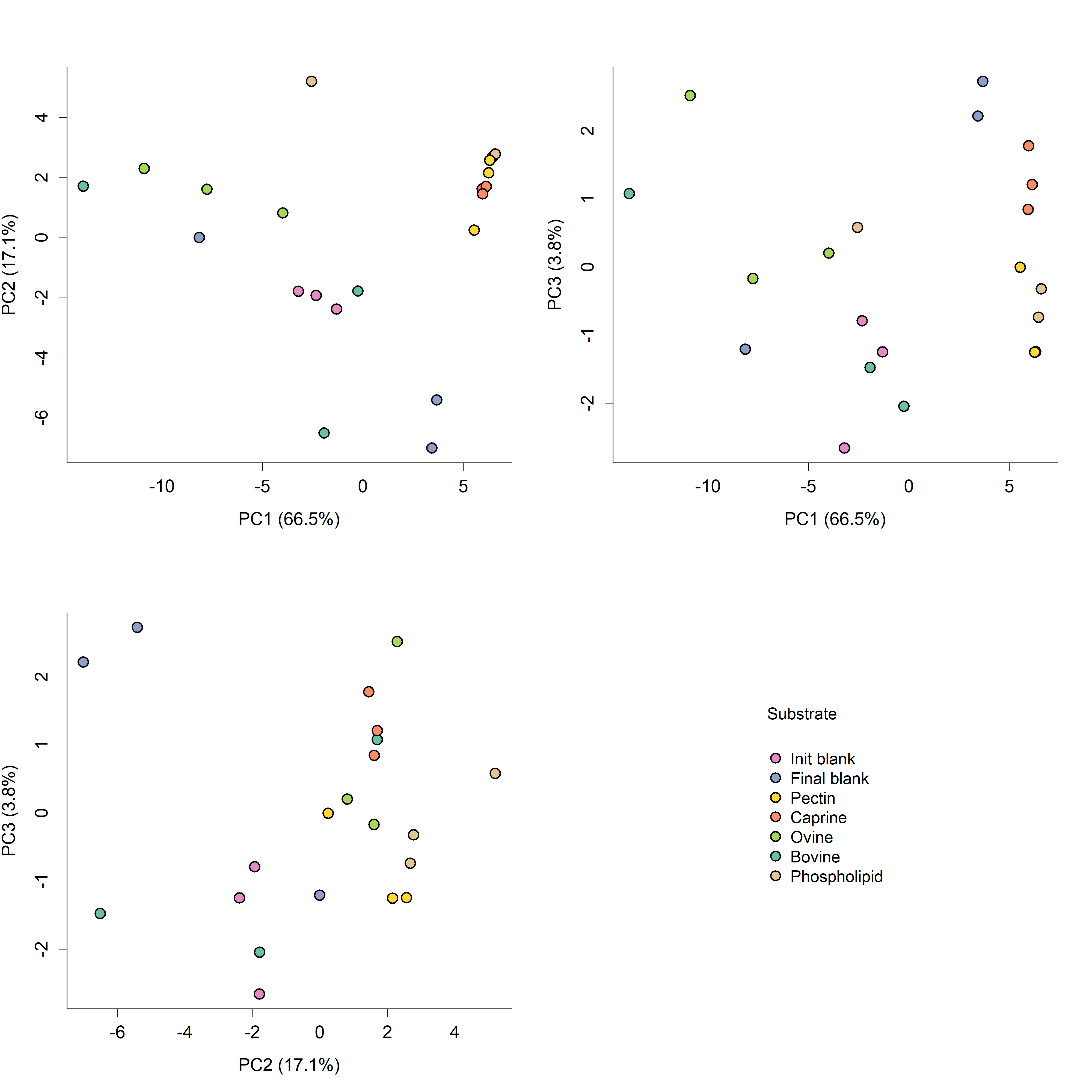


Figure S1. Piglets Ileal and caecal microbiota. Principal Coordinates Analysis (PCoA) plot of unweighted Unifrac phylogenetic distances of the piglets ileal communities after *in vitro* fermentation of digested phospholipid concentrated (PC), bovine, caprine and ovine milk fat globule membrane (MFGM).


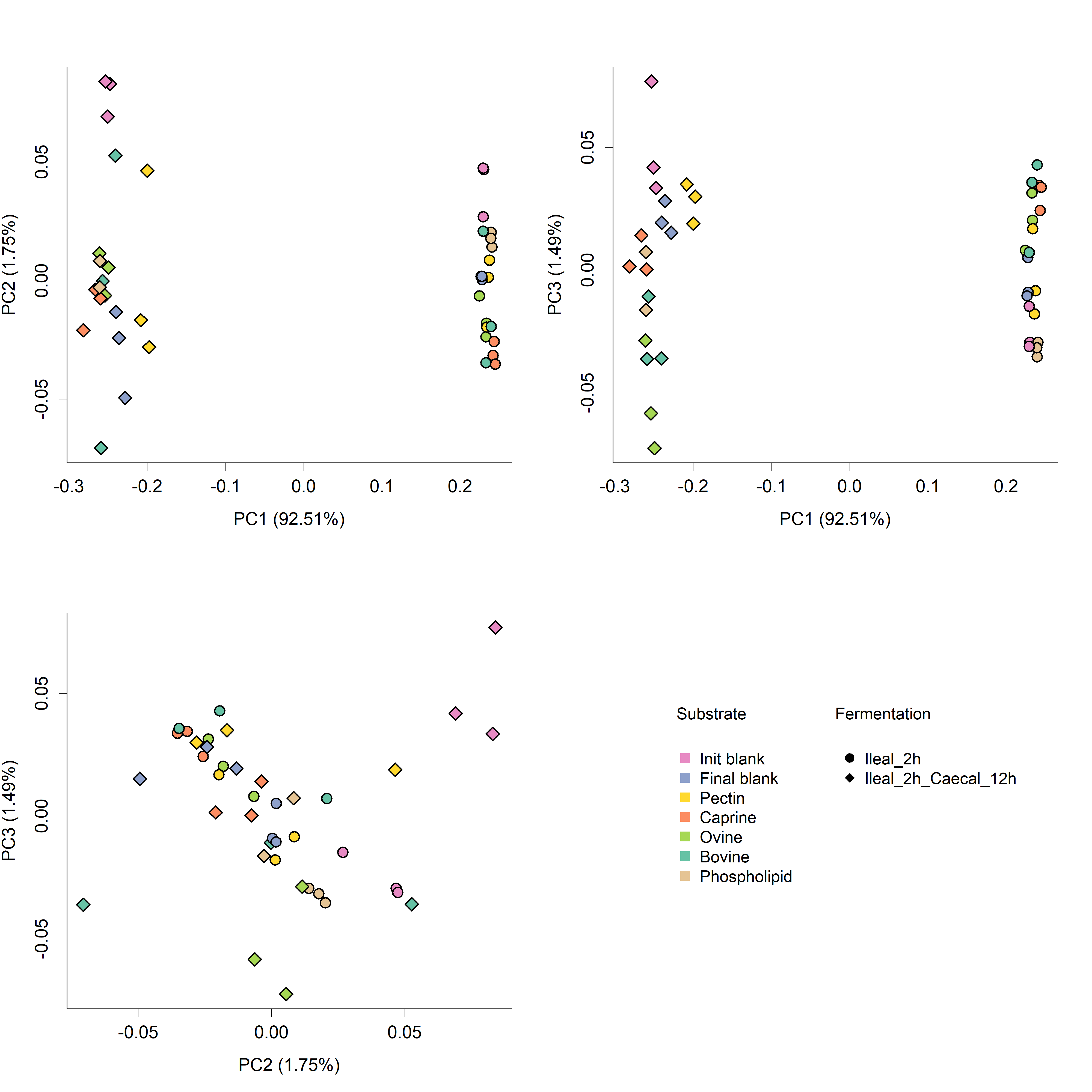


Figure S2. Piglets Ileal and caecal microbiota. Principal Coordinates Analysis (PCoA) plot of unweighted Unifrac phylogenetic distances of the piglets ileal and ileal+ caecal communities after *in vitro* fermentation of digested phospholipid concentrated (PC), bovine, caprine and ovine milk fat globule membrane (MFGM).


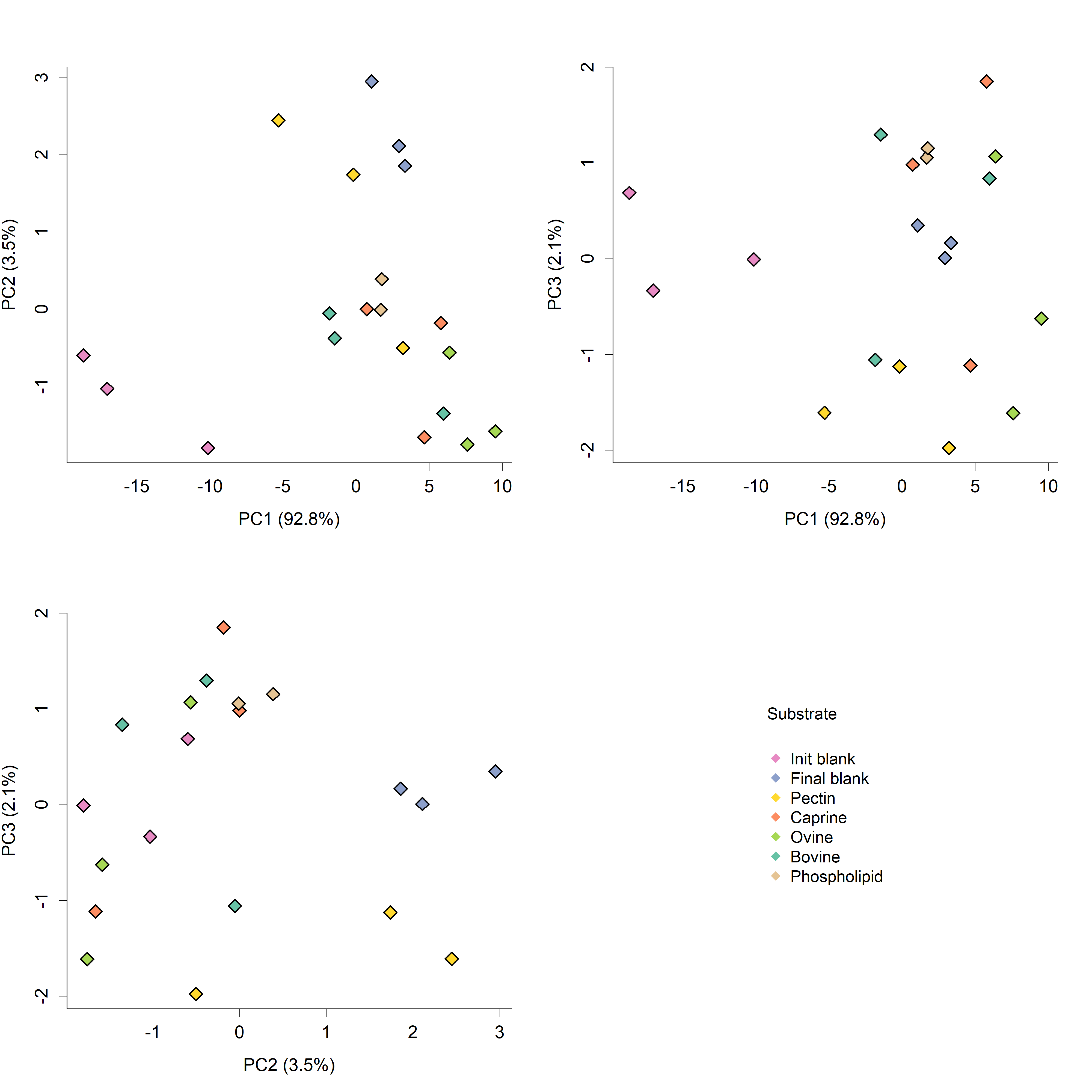


Figure S3. Piglets Ileal and caecal microbiota. Principal Coordinates Analysis (PCoA) plot of unweighted Unifrac phylogenetic distances of the piglets ileal+caecal communities after *in vitro* fermentation of digested phospholipid concentrated (PC), bovine, caprine and ovine milk fat globule membrane (MFGM).
